# A different immunologic profile characterizes patients with HER-2-overexpressing and HER-2-negative locally advanced breast cancer: implications for immune-based therapies

**DOI:** 10.1186/bcr3060

**Published:** 2011-11-23

**Authors:** Elena Muraro, Debora Martorelli, Elisa Turchet, Gianmaria Miolo, Simona Scalone, Elisa Comaro, Renato Talamini, Katy Mastorci, Davide Lombardi, Tiziana Perin, Antonino Carbone, Andrea Veronesi, Diana Crivellari, Riccardo Dolcetti

**Affiliations:** 1 Cancer Bio-Immunotherapy Unit, Centro di Riferimento Oncologico, IRCCS - National Cancer Institute, via Franco Gallini 2, Aviano (PN), 33081, Italy; 2 Division of Medical Oncology C, Centro di Riferimento Oncologico, IRCCS - National Cancer Institute, via Franco Gallini 2, Aviano (PN), 33081, Italy; 3 Epidemiology and Biostatistics Unit, Centro di Riferimento Oncologico, IRCCS - National Cancer Institute, via Franco Gallini 2, Aviano (PN), 33081, Italy; 4 Division of Pathology, Centro di Riferimento Oncologico, IRCCS - National Cancer Institute, via Franco Gallini 2, Aviano (PN), 33081, Italy

**Keywords:** Breast cancer, neoadjuvant therapy, HER2, trastuzumab, antitumor immune responses, tumor-associated antigens

## Abstract

**Introduction:**

The clinical efficacy of trastuzumab and taxanes is at least partly related to their ability to mediate or promote antitumor immune responses. On these grounds, a careful analysis of basal immune profile may be capital to dissect the heterogeneity of clinical responses to these drugs in patients with locally advanced breast cancer undergoing neoadjuvant chemotherapy.

**Methods:**

Blood samples were collected from 61 locally advanced breast cancers (36 HER2^- ^and 25 HER2^+^) at diagnosis and from 23 healthy women. Immunophenotypic profiling of circulating and intratumor immune cells, including regulatory T (Treg) cells, was assessed by flow cytometry and immunohistochemistry, respectively. Serum levels of 10 different cytokines were assessed by multiplex immunoassays. CD8^+ ^T cell responses to multiple tumor-associated antigens (TAA) were evaluated by IFN-γ-enzyme-linked immunosorbent spot (ELISPOT). The Student's *t *test for two tailed distributions and the Wilcoxon two-sample test were used for the statistical analysis of the data.

**Results:**

The proportion of circulating immune effectors was similar in HER2^+ ^patients and healthy donors, whereas higher percentages of natural killer and Treg cells and a lower CD4^+^/CD8^+ ^T cell ratio (with a prevalence of naïve and central memory CD8^+ ^T cells) were observed in HER2^- ^cases. Higher numbers of circulating CD8^+ ^T cells specific for several HLA-A*0201-restricted TAA-derived peptides were observed in HER2^+ ^cases, together with a higher prevalence of intratumor CD8^+ ^T cells. Serum cytokine profile of HER2^+ ^patients was similar to that of controls, whereas HER2^- ^cases showed significantly lower cytokine amounts compared to healthy women (IL-2, IL-8, IL-6) and HER2^+ ^cases (IL-2, IL-1β, IL-8, IL-6, IL-10).

**Conclusions:**

Compared to HER2- cases, patients with HER2-overexpressing locally advanced breast cancer show a more limited tumor-related immune suppression. This may account for the clinical benefit achieved in this subset of patients with the use of drugs acting through, but also promoting, immune-mediated effects.

## Introduction

Preoperative or neoadjuvant chemotherapy (NC) is currently considered to be the standard of care for locally advanced and inoperable breast cancer. One of the main advantages of this approach is the reduction of tumor size, which increases the possibility of performing smaller resections of operable tumors with better cosmetic outcomes [1,2]. Other potential benefits of NC include an early assessment of response to chemotherapy and the possibility of obtaining prognostic/predictive information, based on the pathologic response to therapy [3].

Although breast cancers that overexpress human epidermal growth factor receptor-2 (HER2) are characterized by a poor prognosis [4,5], higher rates of complete responses are currently achieved in HER2^+ ^patients by standard chemotherapy, mainly in association with trastuzumab [6,7], in comparison with HER2^- ^patients. Like other monoclonal antibodies used in anticancer therapy, the activity of trastuzumab is largely dependent on immuno-mediated mechanisms. In fact, besides triggering antibody-dependent cytotoxicity (ADCC), trastuzumab also enhances HLA class I-restricted presentation of endogenous HER2 antigen via the proteasome pathway, and sensitizes HER2-overexpressing tumors to killing by MHC class I-restricted HER2-specific cytotoxic T lymphocytes (CTLs) [7]. Intriguingly, other drugs used in NC regimens have also been shown to enhance antigen-specific immune responses in both *in vitro *and animal models. In particular, taxanes have immunostimulatory effects against tumor cells and suppress cancer not only through inhibition of cell division [8,9]. Indeed, hosts immune functions are highly enhanced after docetaxel treatment [10], and paclitaxel plays a positive role in controlling tumor growth, probably through the induction of IL-8 [8]. Furthermore, taxanes induce macrophage-mediated tumor death, stimulate the production of pro-inflammatory cytokines (TNF-α, IL-12, and IL-1), and increase lymphokine activated killer (LAK) cell and natural killer (NK) cell antitumor activity [10,11].

Given the evidence that tumor cells may be immunogenic, more than 60 TAAs have been identified and, as observed for other tumors, breast cancer cells were also shown to express TAAs [12,13]. Moreover, convincing data demonstrate that spontaneous antitumor responses to TAAs may harness host's immune system to fight against cancer, underscoring the need of a retained or only minimally compromised immunological proficiency particularly in patients treated with chemotherapeutic regimens including immunomodulating drugs. Nevertheless, only limited information is available on the extent of spontaneous T cell responses to breast cancer-associated antigens in patients with locally advanced tumors.

Considering that breast cancer patients may show different types and extent of tumor-related immune dysfunctions [11,14,15], we reasoned that the efficiency of the host immune system could influence the responses to current NC regimens. Therefore, in the present study, we have carried out an extensive immunologic profiling of patients with locally advanced breast cancer at the time of diagnosis, as a first step towards a better understanding of the possible role of antitumor immune responses in mediating the clinical outcome of NC. The results presented herein demonstrate that patients with HER2^+ ^and HER2^- ^breast cancer have a different basal immunologic profile. In particular, our data are consistent with a more limited tumor-related immune suppression in patients with HER2-overexpressing tumors, an observation that may at least in part account for the clinical benefit achieved in this subset of patients by drugs acting through immune-mediated effects.

## Materials and methods

### Patients and healthy donors

Our analysis included 61 patients with histologically confirmed locally advanced breast carcinoma (defined as not susceptible of conservative surgery at diagnosis; UICC, International Union Against Cancer, stage II to III; Table 1). *HER2 *status was assessed by immunohistochemistry and chromogenic *in situ *hybridization (CISH) or fluorescence *in situ *hybridization (FISH) in the case of IHC 2+. All patients had the following clinical features: Eastern Cooperative Oncology Group performance status of 0 or 1; baseline left ventricular ejection fraction measured by ultrasonography greater than 50%; adequate organ function (bone marrow function: neutrophils ≥2.0 × 10^9^/L, platelets ≥120 × 10^9^/L; liver function: serum bilirubin <1.5 times the upper limit of normal (ULN), transaminases <2.5 times ULN, alkaline phosphatase ≤2.5 times ULN, serum creatinine <1.5 times ULN). This study was carried out according to the ethical principles of the Declaration of Helsinki and approved by the local ethics committee. All patients gave written informed consent. Heparinised blood and sera were also collected from 23 age-matched healthy women as controls. Patient's and donor's HLA genotyping was performed by PCR sequencing based typing with primers specific for both locus A and B [16]. Peripheral blood mononuclear cells (PBMCs) were freshly isolated from heparinised blood of patients or healthy donors by Ficoll-Hypaque gradient (Lymphoprep, Fresenius Kabi Norge Halden, Norway) using standard procedures and viably frozen at -180°C until use. Serum samples were obtained with blood centrifugation at 2,100 rpm and maintained at -80°C.

**Table 1:** Patients' characteristics

Characteristic		
**Mean age, years**	44.7	46.3
Range	23-70	32-67
**Hormone receptor status^1^**		
ER+ and PgR+	6	23
ER+ and PgR-	4	5
ER- and PgR-	13	7
ER- and PgR+	2	1
**HER2/neu**		
0-1		27
2+		9
CISH/FISH not amplified		9
CISH/FISH amplified		/
3+	25	
**Tumor size**		
T2	18 (2 multifocal)	26 (2 multifocal)
T3	7 (1 multifocal)	10 (1 multifocal)
**Clinical lymph node involvement**		
N +	23	23
N -	2	13
**Histotype**		
ductal	24	26
lobular	/	7
others	1	3
**Grading^2^**		
G1	/	/
G2	4	18
G3	17	15
G(x)		1
NA	4	2

^1 ^Hormone receptor status is significantly different between HER2+ and HER2- patients, with HER2-overexpressing cancers being mostly ER- and PgR- (*P *= 0.01).

CISH, chromogenic *in situ *hybridization; ER, estrogen receptor; FISH, fluorescence *in situ *hybridization; HER-2, human epidermal growth factor receptor-2; PgR, progesterone receptor.

^2 ^Grading is significantly different between the two cohorts of patients: HER2+ patients are mainly G3, while HER2- patients are mostly G2 (*P *= 0.01).

### Peptide selection and synthesis

A total of 13 immunogenic HLA-A*0201 nonamer (9-mer) peptides, derived from different breast cancer-associated antigens (survivin, mammaglobin-A, HER2, mucin-1, taxol-resistence associated gene 3, and bcl-XL) were selected for the study. The HLA-A*0201-restricted Flu matrix 1 (M158-66) peptide (GILGFVFTL) was used as the positive control. All peptides were produced by fluorenylmethoxycarbonil synthesis (Primm, Milan, Italy) and purity (>95%) was determined by reverse-phase high-performance liquid chromatography and verified by mass spectral MALDI-TOF analysis. Peptides were dissolved in DMSO at a concentration of 2.5 mg/ml and stored at -70°C until use. Work stocks for each peptide were prepared in PBS at a final concentration of 500 μg/ml and stored frozen.

### Flow cytometry

The following fluorescent-conjugated monoclonal antibodies were used: α-CD3 fluorescein isothiocyanate (FITC) or phycoerythrin-texasred (ECD; mouse immunoglobulin (Ig) G1, clone UCHT1), α-CD4 phycoerythrin-cyanine5 (PC5; mouse IgG1, 13B8.2), α-CD8 phycoerythrin-cyanine7 (PC7; mouse IgG1, SFCI2IThy2D3), α-CD16 FITC (mouse IgG1, 3G8), α-CD19 FITC (mouse IgG1 k, J3-119), α-CD25 ECD (mouse IgG2a, B1.49.9), and α-CD45RA ECD (mouse IgG1, 2H4LDH11LDB9) all from Beckman Coulter (Fullerton, CA, USA); α-CD56 phycoerythrin (PE; mouse IgG1 k, B159) and α-CD197 PE (CCR7, rat IgG2a k, 3D12) purchased from BD Biosciences (Becton Dickinson, Franklin Lakes, NJ, USA); α-CD4 PC7 (mouse IgG1 k, RPA-T4), α-CD127 PC5 (mouse IgG1, eBioRDR5), and α-FoxP3 PE (Rat IgG2a k, PCH101) from eBioscience (San Diego, CA, USA). Properly labelled isotypic antibodies were used as negative controls. All antibodies were used in an appropriate volume of 10% rabbit serum (Dako, Glosdrup, Denmark) and PBS (Biomerieux, Marcy l'Etoile, France) to reduce nonspecific signal. Intracellular FoxP3 was determined using the eBioscience FoxP3 Staining Buffer Set (eBioscience, San Diego, CA, USA) according to the manufacturer's instructions. Briefly, after surface molecules staining, cells were fixed and permeabilized with fixation/permeabilization buffer for 30 minutes at 4°C, washed twice, and labelled with FoxP3 antibody in the presence of 2% rabbit serum in PBS at 4°C for at least 30 minutes and, after two washes, cells were re-suspended in PBS with 1% paraformaldehyde. At least 5 × 10^4 ^cells for surface markers and 1 × 10^6 ^cells for intracellular staining were acquired. Cytofluorimetric analysis was performed with a Cytomics FC500 (Beckman Coulter, Fullerton, CA, USA) and data were analyzed with CXP software (Beckman Coulter, Fullerton, CA, USA).

### IFN-γ ELISPOT assay

The interferon (IFN)-γ release enzyme-linked immunosorbent spot (ELISPOT) assay was performed using a commercial kit (Human IFN gamma ELISPOT; Thermo scientific, Rockford, IL, USA), according to manufacturer's instructions. The assay was carried out using autologous peptide-pulsed monocytes as antigen presenting cells (APCs) and isolated CD8^+ ^T lymphocytes as responders. Monocytes, isolated by a two hour plastic adherence step from patient's PBMCs, were loaded with 10 μg/ml of each 9-mer peptide in complete medium, supplemented with 5 μg/ml of human β2-microglobulin, and incubated for two hours at 37°C with 5% CO_2_. Purified effectors were obtained by immunomagnetic enrichment protocols using the human CD8^+ ^T cell isolation kit II (Miltenyi Biotec, Bergisch Gladbach, Germany), and then cultured with peptide-loaded monocytes (50,000 cells/well) at 1:1 effector:target ratio. FLU M158-66 and unstimulated monocytes were used as positive and negative controls, respectively. Cells were seeded onto ELISPOT capture plates in triplicates and incubated for 48 hours at 37°C with 5% CO_2_. All plates were evaluated by a computer-assisted ELISPOT reader (Eli.Expert, A.EL.VIS GmbH, Hannover, Germany). The number of spots in negative control wells (range of 0 to 5 spots) was subtracted from the number of spots in stimulated wells. Responses were considered significant if a minimum of five IFN-γ producing cells were detected in the wells.

### Cytokine detection

Levels of IL-1α, IL-1β, IL-2, IL-6, IL-8, IL-10, IL-12p70, TNF-α, and granulocyte macrophage colony-stimulating factor (GM-CSF) were evaluated using the SearchLight^® ^multiplex arrays (Food and Drug Administration approved, Aushon Biosystems, TEMA Ricerca, Bologna, Italy) according to the manufacturer's instructions. Briefly, custom human 8-plexarray and human 1-plexarray (for GM-CSF detection) with pre-spotted cytokine-specific antibodies were used. Standards or pre-diluted samples were added in duplicate and, after one hour of incubation at room temperature and three washes, biotinylated antibody reagent was added to each well. After 30 minutes incubation at room temperature and three washes, block solution was added to stabilize the signal. The addition of Streptavidin-HRP Reagent and SuperSignal^® ^Substrate, and the acquisition of luminescent signal with a cooled CCD (Charge Coupled Device) camera, together with data analysis and processing, were performed by TEMA Ricerca laboratories' customer service (Bologna, Italy).

Transforming growth factor (TGF)-β1 serum levels were assessed through DRG TGF-β1 ELISA (DRG Instruments GmbH, Marburg, Germany) under instructions. Pre-diluted samples and standards underwent appropriate acidification and neutralization before testing. Briefly, pre-treated standards, controls and samples were dispensed into wells in duplicate and plates were incubated overnight at 4°C. After three washes, antiserum was added to wells and incubated for 120 minutes at room temperature, plate was rinsed three times and anti-mouse biotin (enzyme conjugate) was dispensed and incubated for 45 minutes. After three washes, enzyme complex was added to wells, then plates were incubated 45 minutes and washed three times. After the addition of substrate solution for 15 minutes, the reaction was stopped and the adsorbance at 450 ± 10 nm was determined with a microtiter plate reader (Bio-Tek Instruments, Winooski, VT, USA). As there is an extremely variable range of normal values reported in the literature, healthy women serum levels were taken as references.

### Immunohistochemistry

Considering that the diagnostic biopsy is not fully representative of the whole tumor mass, the lymphoid infiltrate was investigated in 40 primary advanced breast carcinomas from a separate series of patients who underwent surgical resection during the past decade at our institution. Twenty cases were HER-2^+ ^(3+) and 20 HER-2^- ^(0 or 1+). All specimens were routinely fixed in 10% buffered formalin, embedded in paraffin and then stained with H&E for histological examination. For immunohistochemical analyses, 2 to 3 µm serial sections of primary tumors were processed with automated immunostainer Benchmark XT (Ventana, Tucson, AZ, USA), and staining was carried out with the following antibodies: CD8 (clone SP57, Ventana Medical System, Tucson, AZ, USA); FoxP3 (clone 259D/C7, BD Pharmingen, Franklin Lakes, NJ, USA) diluted 1:100 e TiA-1 (clone TiA-1, Bioreagents, Golden, CO, USA) diluted 1:100. Nuclear counterstaining was accomplished with Harris' hematoxylin. Omission of the primary antibody was used as a negative control. The results for staining were evaluated with reference to the number of unequivocally stained lymphoid cells. Ten randomly chosen representative microscopic fields were counted at 40x original magnification.

### Statistical analysis

Chi-square test was used to compare hormone receptor (HR) expression and grading within HER2^- ^and HER2^+ ^populations. Data obtained from multiple independent experiments were expressed as mean and standard deviation for immunophenotypic analysis and ELISPOT assays; cytokine box plots were obtained with SigmaPlot. The Student's *t *test for two tailed distributions and the Wilcoxon two-sample test were used for the statistical analysis of the data. Odds ratio and 95% confidence intervals in multivariate analysis were performed to assess the possible influence of clinico-pathological variables on the immunological correlations observed: immunological variables (cytokine levels) were divided into quartiles according to their concentrations and then stratified for HR expression (estrogen receptor (ER)- progesterone receptor (PgR)- and ER+ and/or PgR+), and tumor grading (G2 or G3). Wilcoxon rank-test was used to compare the distribution of intratumor CD8^+ ^and FoxP3^+ ^cells between HER2^+ ^and HER2^- ^cases. Results were considered to be statistically significant when *P *≤ 0.05 (two-sided).

## Results

### HER2^+ ^and HER2^- ^patients exhibit a different distribution of circulating and intratumor immune cells

**Figure 1:**
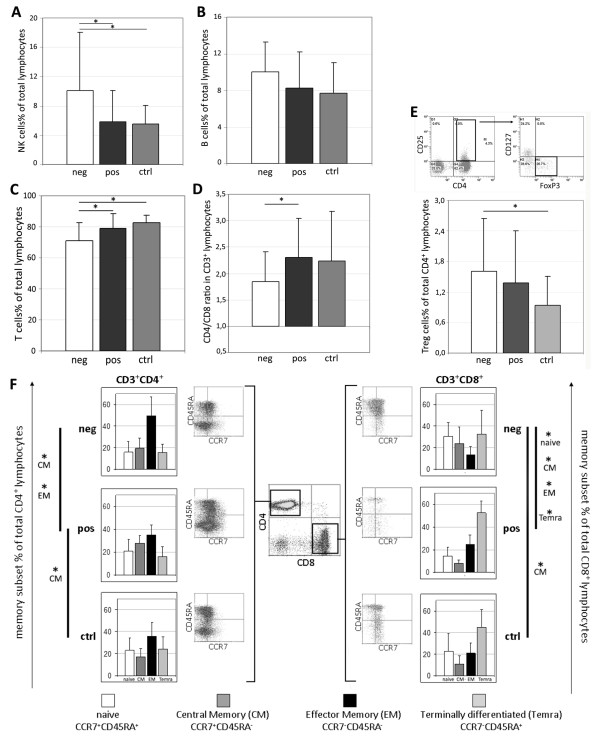
**Immunophenotypic characterization of peripheral blood lymphocytes**. Percentages of (**a**) natural killer cells (NK; CD3^-^CD16^+^CD56^+^; neg = 20, pos = 17, ctrl = 17), (**b**) B cells (CD3^-^CD19^+^; neg = 14, pos = 15, ctrl = 15), (**c**) T lymphocytes (CD3^+^CD19^-^; neg = 20, pos = 17, ctrl = 17), (**d**) CD4^+^/CD8^+ ^ratio (CD3^+^CD4^+^/CD3^+^CD8^+^; neg = 20, pos = 17, ctrl = 17), and **(e) **regulatory T cells (Treg; CD3^+^CD4^+^CD25^high^CD127^low^FoxP3^+^; neg = 20, pos = 15, ctrl = 17) assessed in HER2^- ^(*neg*), HER2-overexpressing (*pos*) patients and age-matched healthy women (*ctrl*). (**f**) Differentiation (memory) status of CD3^+^CD4^+ ^and CD3^+^CD8^+ ^lymphocytes was investigated through CCR7 and CD45RA expression (neg = 12; pos = 10; ctrl = 10). Statistical analysis was performed with t Student test. *, *P*<0.05 (refer to text for exact *P *value). HER-2, human epidermal growth factor receptor-2.

The distribution of different circulating immune populations was investigated at diagnosis by multiparametric flow cytometry comparing 17 women with HER2-overexpressing cancers, 20 women with HER2^- ^tumors, and 17 healthy women, who were considered as controls. A significantly lower percentage of CD3^+ ^T cells (Figure 1c) was observed in HER2^- ^patients with respect to both HER2^+ ^cases (*P *= 0.028) and controls (*P *= 0.0003). In parallel, among the CD3^- ^cell populations, higher numbers of CD16^+^CD56^+ ^NK cells were detected in HER2^- ^cases compared with HER2^+ ^patients (*P *= 0.049) and healthy donors (*P *= 0.025; Figure 1a). Interestingly, no major difference in the distribution of circulating CD3^- ^cells was observed between HER2-overexpressing patients and controls. The percentage of B cells (Figure 1b) was not significantly different among the three groups investigated, even if HER2^- ^(n = 14) patients showed slightly higher numbers of CD3^-^CD19^+ ^cells than HER2^+ ^patients (n = 15) and donors (n = 13; *P *= 0.07).

When the CD3^+ ^population was considered separately, HER2^- ^patients showed significantly higher percentages of CD8^+ ^T cells (*P *= 0.028; data not shown) and a lower CD4^+^/CD8^+ ^ratio (Figure 1d; *P *= 0.046) when compared with HER2^+ ^cases. Because of the different contribution of memory subsets in mediating antitumor immune responses [17-19], the differentiation state of T cell was investigated through the combined analysis of the chemokine receptor CCR7 and the CD45RA isoform, to distinguish CCR7^+^CD45RA^+ ^naïve, CCR7^+^CD45RA^- ^central memory (CM), CCR7^-^CD45RA^- ^effector memory (EM), and CCR7^-^CD45RA^+ ^terminally differentiated (Temra) cells [20]. Although the three studied groups showed a similar distribution of memory CD3^+ ^T cell subsets (not shown), separate analysis of the CD4^+ ^and CD8^+ ^compartments disclosed remarkable differences. In fact, compared with controls, HER2^+ ^patients carried a higher percentage of CM CD4^+ ^T cells (*P *= 0.003), whereas HER2^- ^cases showed significantly higher numbers of CM CD8^+ ^T lymphocytes (*P *= 0.022). Moreover, a higher percentage of EM CD4^+ ^cells (*P *= 0.023), at the expense of the CM subset (*P *= 0.023), was found in HER2^- ^cases compared with HER2^+ ^patients (Figure 1f). Finally, the two groups of breast cancer patients showed a completely different memory sharing among CD8^+ ^cells, with a prevalence of naïve (*P *= 0.002) and CM cells (*P *= 0.005) in HER2^- ^cases and higher percentages of EM (*P *= 0.005) and Temra cells (*P *= 0.012) in HER2^+ ^patients.

Several studies argued the unfavorable involvement of circulating regulatory T cells (Tregs) in cancer progression, demonstrating the presence of increased numbers of CD4^+^CD25^high^FoxP3^+ ^especially in metastatic cancers [21]. Considering that this immunophenotypic characterization is unsuitable for uniquely defining this specialized T cell subset, we used IL-7 receptor (CD127) down-regulation as a further feature indicative of suppressive functions [22] and identified Tregs as CD4^+^CD25^high^CD127^low^FoxP3^+ ^cells. The analysis showed that total numbers of so determined Tregs were not significantly different between patients and controls. Nevertheless, considering the two groups of patients separately, HER2^- ^exhibited a significantly higher percentage of circulating Treg cells (*P *= 0.02) when compared with healthy donors (Figure 1e).

**Figure 2:**
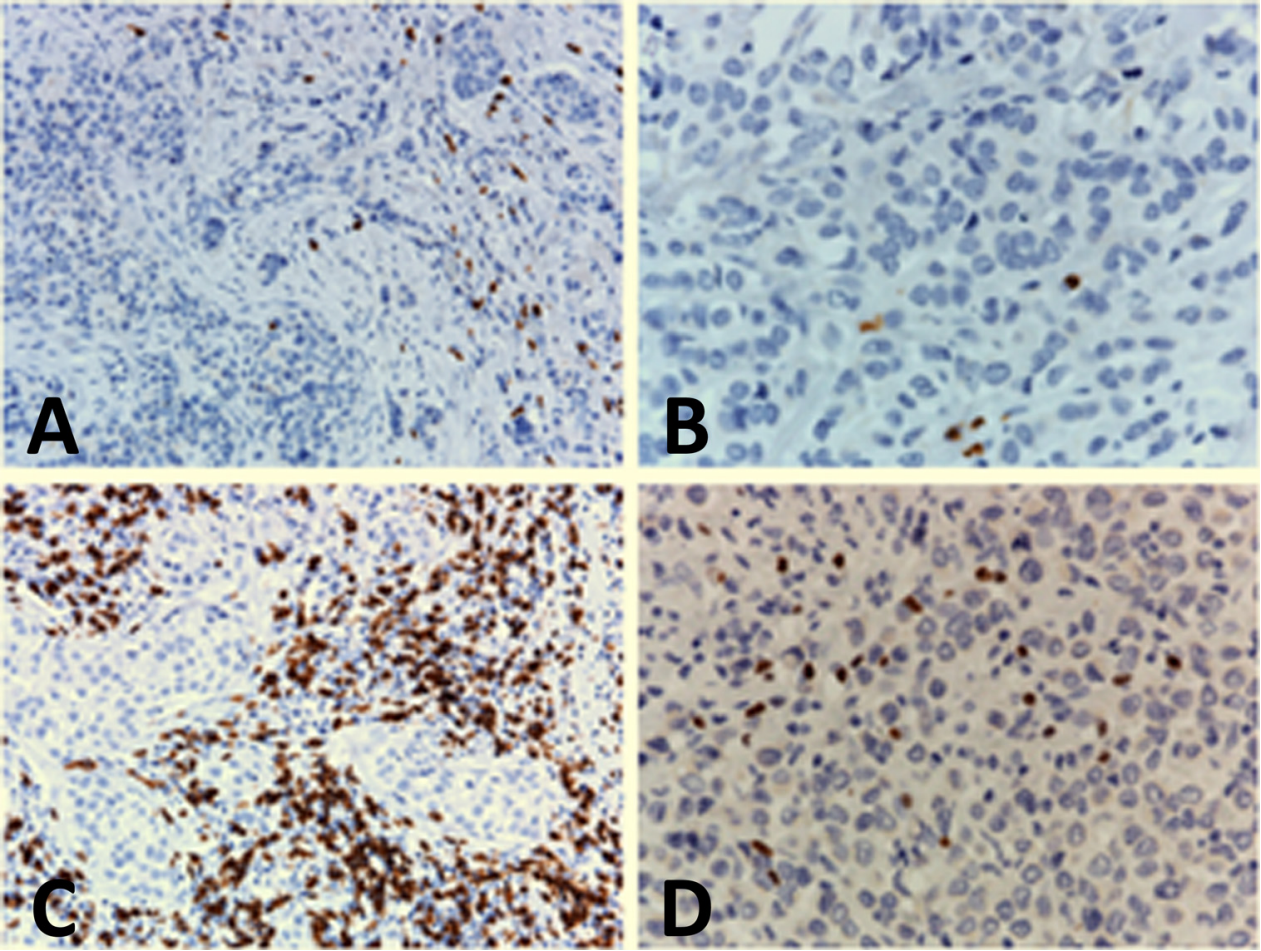
**Composite figure showing CD8 and FoxP3 expression in lymphoid cells infiltrating representative HER2^- ^or HER2^+ ^breast carcinomas**. (**a**) Few CD8^+ ^cells are present within the tumor, and infiltrate tumor nests in a HER2^- ^case. **(b) **Some FoxP3^+ ^cells infiltrate a HER2^- ^breast carcinoma. **(c) **CD8^+ ^cells are numerous and surround tumoral cords in a HER2^+ ^breast carcinoma. **(d) **Higher numbers of FoxP3^+ ^cells infiltrate a HER2^+ ^case. **(a to d) **Immunohistochemical stain; paraffin section; Hematoxylin counterstain; **(a and c) **20× original magnification, **(b and d) **40× magnification. HER2: human epidermal growth factor receptor-2.

Characterization of the lymphoid infiltrate in an unrelated series of locally advanced breast cancers disclosed a significantly higher prevalence of intratumor CD8^+ ^T cells in HER2^+ ^cases (median 1000, range: 730 to 1880) as compared with the HER2^- ^subgroup (median 234, range: 117 to 890, *P *= 0.04). TiA-1^+ ^cells were also more abundant in HER2^+ ^tumors and only rarely detected in the HER2^- ^subgroup (not shown). Conversely, the median numbers of FoxP3^+ ^cells was higher in HER2^+ ^cases (170, range: 50 to 508) than in HER2^- ^tumors (25, range: 10 to 108, *P *= 0.04; Figure 2).

### HER2^+ ^patients display enhanced CD8^+ ^T cell responses to different TAA-derived epitopes compared with both HER2^- ^patients and healthy donors

**Figure 3:**
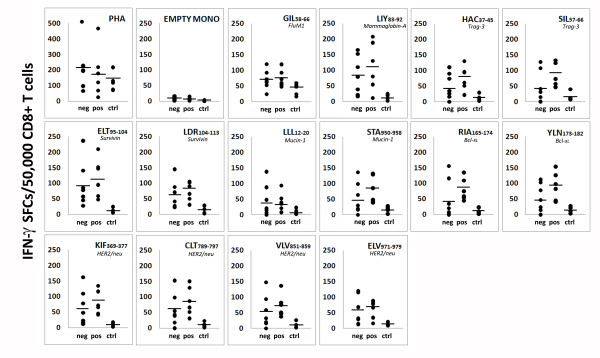
**CD8^+ ^T cell responses to multiple breast cancer-associated antigenic epitopes assessed by IFN-γ-ELISPOT (interferon-γ-Enzyme Linked Immunosorbent Spot)**. All tests were performed using CD8^+ ^purified T cells as effectors and autologous peptide-loaded monocytes as antigen presenting cells (APCs; effector:target ratio of 1:1). The number (enumerated as SFC, spot forming cells) of TAA-specific (or FluM1-specific, flu matrix protein1-derived epitope) circulating CD8^+ ^T cells was investigated in HER2^- ^(neg = 7) and HER2^+ ^(pos = 6) breast cancer patients, whereas antigen-specific responses of healthy women were used as controls (ctrl = 5). PHA-loaded and empty monocytes (EMPTY MONO) were used as positive and negative controls, respectively. For peptides amino acid sequences, refer to Table 2. HER-2, human epidermal growth factor receptor-2; TAA, tumor-associated antigens.

Spontaneous CD8^+ ^T cell responses to 13 TAA-derived peptides (Her2, muc-1, mam-A, trag-3, survivin, bcl-x_L_; Table 2) were evaluated by IFN-γ ELISPOT assay in six HER2^+ ^and seven HER2^- ^HLA-A*0201^+ ^patients and five HLA-A*0201^+ ^age-matched healthy women. IFN-γ-secreting CD8^+ ^T cells were detected in all samples (Figure 3), although higher numbers of CD8^+ ^T cells specific for all epitope peptides investigated were observed in both HER2^+ ^and HER2^- ^patients compared with healthy donors (both *P*<0.002). Notably, the number of circulating TAA epitope-specific CD8^+ ^T cells was higher in HER2^+ ^cases compared with HER2^- ^(*P*<0.005), particularly against peptides derived from trag-3, muc-1, and bcl-x_L _(Figure 3). Empty monocytes were considered as negative controls and the number of spots was usually at the background level (<10 SFC/50,000 CD8^+ ^cells). No significant differences were found between patients and donors against Flu M1 GIL_58-66 _peptide, used as positive control (49<SFC/50,000 CD8+ cells>76), and similar levels of responses were also observed against PHA (167<SFC/50,000 CD8+ cells>221), confirming a retained T cell responsiveness.

**Table 2:** Library of immunogenic peptide epitopes used to evaluate CD8+ T cell responses to breast-cancer-associated antigens

Mammaglobin-A	LIY_83-92_	LIYDSSLCDL	*34*
Trag-3	**HAC_37-45_**	HACWPAFTV	*35*
	**SIL_57-66_**	SILLRDAGLV	*35*
Survivin	**ELT_95-104_**	ELTLGEFLKL	*36*
	**LDR_104-113_**	LDRERAKNKI	*36*
Mucin-1	**LLL_12-20_**	LLLLTVLTV	*33*
	**STA_950-958_**	STAPPVHNV	*33*
Bcl-xL	**RIA_165-174_**	RIAAWMATYL	*13*
	**YLN_173-182_**	YLNDHLEPWI	*13*
Her2/neu	**KIF_369-377_**	KIFGSLAFL	*12*
	**CLT_789-797_**	CLTSTVQLV	*12*
	**VLV_851-859_**	VLVKSPNHV	*12*
	**ELV_971-979_**	ELVSEFSRM	*12*

HER-2, human epidermal growth factor receptor-2; Trag-3, taxol-resistance associated gene 3.

### HER2^+ ^patients show similar serum cytokine profile in respect to donors

**Figure 4:**
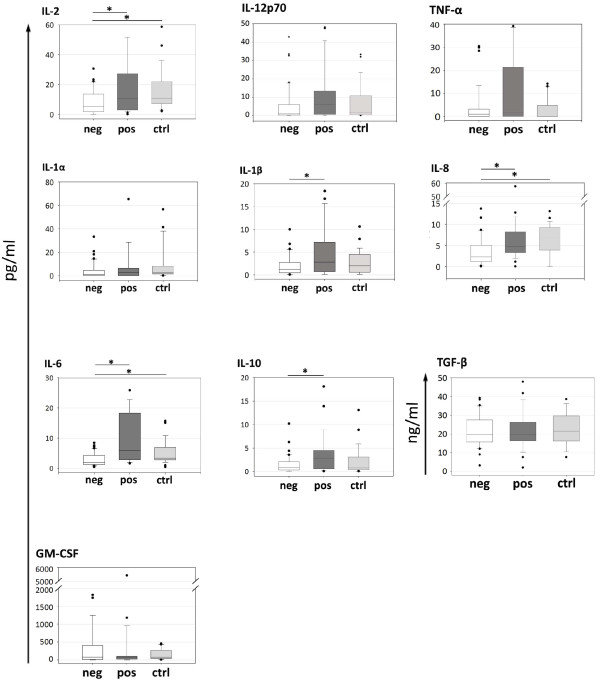
**Serum cytokine profile**. Interleukin (IL)-2, IL-12p70, IL-1α, IL-1β, IL-8, IL-6, IL10 (neg = 36, pos = 25, ctrl = 23), Tumor necrosis factor-α (TNF-α; neg = 33, pos = 23, ctrl = 23), granulocyte-macrophage colony-stimulating factor (GM-CSF; neg = 33, pos = 23, ctrl = 19) and transforming growth factor-β (TGF-β; neg = 27, pos = 21, ctrl = 9) levels were evaluated in serum samples from HER2^- ^(neg), HER2^+ ^(pos) patients and age-matched healthy women (ctrl). Statistical analysis was performed with the Wilcoxon two-sample test. *, *P*<0.05 (refer to text for exact *P *value). HER2, human epidermal growth factor receptor-2.

The serum levels of 10 different cytokines were evaluated in all 61 patients and 23 healthy women, considered as internal reference values. No significant difference was observed when comparing the global cohort of patients with controls. However, when breast cancer patients were considered as two different groups based on HER2 expression, the comparison revealed that HER2^- ^patients carried significantly lower amounts of IL-2 (*P *= 0.0222), IL-8 (*P *= 0.009), and IL-6 (*P *= 0.016) with respect to donors (Figure 4), whereas the cytokine profile of HER2^+ ^cases was almost superimposable to that of healthy women (Figure 4). Notably, the most evident differences emerged from the comparison between the two groups of patients, with HER2^- ^cases showing significantly reduced levels of IL-2 (*P *= 0.0229), IL-1β (*P *= 0.0207), IL-8 (*P *= 0.007), IL-6 (*P *= 0.0001), and IL-10 (*P *= 0.0247; Figure 4), independently by other clinical-pathological parameters such as HR expression and tumor grading (*P*-trend in multivariate analysis: IL-1β *P *= 0.002, IL-2 *P *= 0.004, IL-6 *P *= 0.004, IL-8 *P *= 0.02, IL-10 *P *= 0.02; Table 3). No differences were found in IL-1α, TNF-α, IL-12p70, GM-CSF, and TGF-β serum concentrations.

**Table 3:** Odds ratio (OR) and 95% confidence interval (CI) adjusted for hormone receptor expression and tumor grading according to cytokines levels in HER+ and HER- patients

	Cytokines level quartiles^1^				
	1-low	2	3	4-high	*P *trend
**IL-1b**					
N. pos/neg^2^	4/11	5/10	5/10	11/5	
OR (95% CI)	1^3^	6.3 (0.7-53.2)	33.6 (2.3-494.6)	55.4 (4.1-746.0)	***0.002***
**IL-2**					
N. pos/neg^2^	3/12	6/9	6/10	10/5	
OR (95% CI)	1^3^	15.7 (1.6-150.5)	10.3 (1.2-92.4)	31.8 (3.5-289.6)	***0.004***
**IL-6**					
N. pos/neg^2^	2/15	5/9	7/8	11/4	
OR (95% CI)	1^3^	3.8 (0.5-31.0)	8.2 (1.0-65.4)	21.9 (2.6-185.8)	***0.004***
**IL-8**					
N. pos/neg^2^	2/14	6/9	8/7	9/6	
OR (95% CI)	1^3^	3.7 (0.5-26.0)	5.6 (0.7-42.3)	11.7 (1.4-95.5)	***0.02***
**IL-10**					
N. pos/neg^2^	6/11	3/9	5/10	11/4	
OR (95% CI)	1^3^	0.3 (0.04-2.6)	1.6 (0.2-12.1)	9.6 (1.5-59.0)	***0.02***

^1^Quartiles were: IL-1b: ≤0.5/0.6-1.7/1.8-4.2/≥4.3; IL-2: ≤2.2/2.3-5.8/5.9-15.8/≥15.9; IL-6: ≤1.6/1.7-3.4/3.5-6.5/≥6.6; IL-8: ≤1.7/1.8-3.7/3.8-6.2/≥6.3; IL-10: ≤0.4/0.5-1.4/1.5-3.3/≥3.4 pg/ml.

^2 ^pos, HER2+; neg, HER2-;

^3 ^Reference category.

HER-2, human epidermal growth factor receptor-2; IL, interleukin.

## Discussion

Evidence accumulated so far indicates that the immune system can influence the initiation and development of cancer and it is widely believed that T lymphocytes represent the most potent antitumor effector cells. In this respect, there is an urgent need to develop therapeutic approaches able to preserve or only minimally impair immune functions since ADCC-promoting therapeutic antibodies, such as trastuzumab, and cancer vaccines are being increasingly used as adjuvant and neoadjuvant treatment modalities [23]. Furthermore, also the therapeutic efficacy of some "conventional" drugs, such as doxorubicin and paclitaxel, involves immunomediated mechanisms [11,24]. On these grounds, we considered it clinically relevant to extensively investigate at diagnosis the immunological profile of locally advanced breast cancer patients who are candidates for NC including immunomodulating drugs. The present study provides baseline immunological data that may constitute a reference for an informative monitoring of immune responses during NC (ongoing study). Provided the feasibility of monitoring antitumor responses during therapy and considering that sera from breast cancer patients should represent a valuable discovery tool to identify potential targets involved in breast cancer progression [25], we focused mainly on peripheral blood as the easiest accessible way to detect and measure immune-changes.

It is worth considering that most studies reporting analyses of systemic immunologic parameters in breast cancer patients included extremely variable series of cases, thus obtaining wide ranges of values and often conflicting results. Our study does not suffer from this limitation, being based on a relatively homogeneous group of patients including only locally advanced cancers and excluding metastatic patients, which are the main contributors to outliers [26,27].

Previous data reported a significant increase in circulating B lymphocytes and NK cells in breast cancer patients in comparison with control groups [14], with a lower total number of T lymphocytes [15]. Interestingly, in our study, HER2^+ ^patients retained a normal distribution in NK cells, and T and B lymphocytes if compared with healthy donors, whereas HER2^- ^cases displayed lower percentages of T cells and higher numbers of NK and, to a lesser extent, of B cells. The increased NK cell numbers and activity reported in pre-treated patients were related to time to treatment failure [14] and were proposed to be the result of activation of the innate immunity by the tumor or dependent on a defective regulation of NK cells in these patients [28]. Conversely, Dewan and colleagues observed significantly lower NK cell activity in PBMC from breast cancer patients, as compared with that of healthy individuals. Intriguingly, this defect was more pronounced in HER2^- ^breast cancer patients [15], suggesting an underlying NK dysfunction in this subgroup. Further, we noticed that HER2^+ ^patients showed an increased CD4/CD8 ratio with respect to HER2^- ^cases, a feature previously associated with a better chance of responding to NC [14]. However, opinions regarding which T-cell subset provides the best tumor protection, especially among memory sub-populations, are still controversial. Indeed experimental evidence suggests that central (T_CM_) and effector (T_EM_) memory T cells can each confer a protective advantage [17-19], with T_CM _providing a reservoir of antigen-specific T cells, ready to expand and replenish the periphery upon secondary challenge, and T_EM _displaying a more activated phenotype capable of granzyme B and perforin expression, IFN-γ secretion, and tumor-specific killing *in vitro *[17]. Compared with healthy donors, our data disclosed a higher percentage of CD4^+ ^T_CM _(CCR7^+^CD45RA^-^) in HER2^+ ^patients and higher numbers of CD8^+ ^T_CM _in HER2^- ^cases. This observation may suggest the existence, in both groups, of an active, though predominantly memory T-cell driven, antitumor response which may benefit and respond to recall antigens from a cancer vaccine. Interestingly, in our study the main variations in memory subsets distribution were found between HER2^- ^and HER2^+ ^patients. Although the CD4^+ ^T cell population of HER2^+ ^cases disclosed a favorable shift to the T_CM _phenotype, in these same patients CD8^+ ^T lymphocytes revealed a predominance of T_EM _and terminally differentiated cells. In contrast, HER2^- ^patients exhibit mainly CCR7^+ ^CD8^+ ^T cells (naïve and T_CM_). It should be considered that peripheral CD8^+ ^T cells expressing effector functions against viral [29,30] or tumor antigens [31] are almost uniformly CCR7^- ^and are endowed with full effector capacities, far greater than naïve or T_CM _cells. Moreover, as the differentiation to terminal effector cells is related to increased cytolytic potential of CD8^+ ^T cells, we hypothesize that the extent of maturation might be due to an effective antitumor response [18].

Pre-existing T cell responses to TAA have been reported in patients with solid tumors [32]; however, these responses usually involve a low frequency of antigen-specific T cells, not detectable in the majority of patients [18]. In this regard, literature data on circulating tumor antigen-specific T cells in breast cancer patients are still conflicting, probably because of the predominant focus on single epitopes [18]. Circulating T cells able to recognize CD8^+ ^epitopes of HER2 [12], MUC-1 [33], mammaglobin-A [34], Trag-3 [35], survivin [36], or bcl-x_L _[13] have been described in distinct papers, but the evaluation of multiepitopic antitumor responses is still lacking. We therefore assessed the amount of IFN-γ-secreting CD8^+ ^T cells specific for a broad spectrum of HLA-A*0201 peptides derived from Her2, muc-1, mam-A, trag-3, survivin, and bcl-x_L_. Notably, we found increased IFN-γ release to all screened epitopes in the global cohort of patients if compared with healthy donors, demonstrating the existence of spontaneous T cell responses against multiple TAA in locally advanced breast cancer patients. The ability to stimulate the generation of antitumor CD8^+ ^T cells seemed to be more pronounced in HER2^+ ^cancers, especially towards Her2-, trag-3-, muc-1-, and bcl-x_L_-derived epitopes. This peculiarity may be useful in the design and optimization of vaccine strategies, which could take advantage of host's pre-existing antitumor immune response. Moreover, the increased numbers of TAA-specific circulating CD8^+ ^T cells characterizing HER2^+ ^patients may positively contribute to the clinical efficacy of trastuzumab, which is able to sensitize HER2-overexpressing tumors to the killing by HER2-specific CTLs [7], and may enhance the antigen-specific immune responses promoted by doxorubicin and paclitaxel [37]. Our findings at the systemic level are also consistent with the demonstration of a significantly higher prevalence of CD8^+ ^T lymphocytes infiltrating HER2^+ ^tumors that could contribute to a better clinical outcome [38]. This suggests that HER2 overexpression may be associated with enhanced immunogenicity of tumor cells and/or with a less immunosuppressive microenvironment. Further characterization of the activation state of lymphocytes infiltrating these tumors is, however, required to draw definitive conclusions in this respect.

It is well recognized that tumors may down-regulate the immune response to tumor antigens by inducing several immune suppressor mechanisms, including Treg recruitment. Increased numbers of Tregs have been correlated with greater disease burden and poorer overall survival [39]. In particular, Treg cells are augmented in the peripheral blood and within the tumor microenvironment in patients with breast carcinomas [40]. Our analysis of FoxP3 expression in intratumor lymphocytes disclosed significantly higher prevalence of FoxP3^+ ^cells in HER2^+ ^tumors. This may be a homeostatic consequence of the higher content of infiltrating CD8^+ ^T cells detected in these cancers, or may reflect an active local recruitment of Tregs. It is worth considering in this respect that conflicting results have been reported with regard to Treg frequencies in HER2^- ^versus HER2^+ ^breast cancers [27,41], discrepancies that could be due in part to tumor staging, but also to the different and often partial phenotypic markers used to identify regulatory T cells. This suppressor cell subset is often identified as CD4^+^CD25^high ^cells [27] or as CD4^+^FoxP3^+ ^lymphocytes [41], even if these markers are unable to uniquely define a regulatory T cell phenotype. Therefore, the use of FoxP3 as a single immunohistochemical marker to identify Tregs may have overestimated the number of Tregs in the HER2^+ ^subgroup. In fact, this approach can not discriminate true FoxP3^+ ^Treg cells from T lymphocytes activated by local stimuli and therefore transiently expressing FoxP3 without being endowed of immunosuppressive functions. To overcome these limitations, we have *bona fide *considered as circulating Tregs only cells expressing the CD4^+^CD25^high^CD127^low^FoxP3^+ ^phenotype, as the down-regulation of the IL-7 receptor (CD127) is associated with suppressive functions [22]. This extended phenotypic definition did not disclose significant differences in Treg distribution between the whole group of breast cancer patients and controls, but it revealed higher numbers of circulating Tregs in HER2^- ^patients with respect to donors. Tregs exert their suppressor activity by inhibiting T cell proliferation, NK cell-mediated cytotoxicity [42], and TAA-specific immunity [43]. On these grounds, the nearly physiological number of circulating Tregs displayed by stage II and III HER2^+ ^breast cancer patients may imply a favorable background for NK-involving therapy such as monoclonal antibodies (trastuzumab), and may further benefit from the spontaneous enhanced antitumor T cell responses.

The likely retained immune proficiency of HER2^+ ^patients is supported by an apparently unchanged cytokine profile, as sharpened by the comparison with serum cytokine levels of healthy women, which displays no significant differences. It is widely accepted that solid tumors are associated with a pathologic shift toward the T-helper type 2 cytokine pattern; whereas T-helper 1-induced inflammation inhibits tumor growth. In breast cancer patients, depressed serum levels of IL-2, GM-CSF, IFN-γ and enhanced TNF-α and IL-6 amounts were reported in comparison with controls [11] and some of these immune dysfunctions are also present in early-stage tumors. In our study, we noticed significantly lower levels of IL-2 and IL-8, but also of IL-6 in HER2^- ^patients with respect to healthy women. Increased serum levels of IL-6 were previously reported in progressive recurrent breast cancer patients [44], especially in the presence of metastasis [26], conversely no metastatic cases were enrolled in our cohort. Interestingly, the comparison of cytokine layouts between HER2^- ^and HER2^+ ^patients disclosed pathogenically relevant differences between the two groups. In particular, HER2^- ^patients showed lower levels of IL-2, previously associated with relapse of the disease [45,46], reduced amounts of the pro-inflammatory cytokines IL-1β and IL-6 (repressors of *in vitro *cell cycle progression) [47], and reduced levels of IL-8, recently reported also in the *in vitro *comparison of HER2^- ^and HER2^+ ^breast cancers cell lines and in serum samples from metastatic breast cancer patients [25]. Finally, the pleiotropic cytokine IL-10, which may exert tumor-promoting activity or considerable antitumor effects, at low and high concentrations, respectively [46], was detected at lower amounts in HER2^- ^patients. On the other hand, the higher levels of IL-2 in HER2^+ ^patients may be consistent with an activation of T cells by TAA-derived peptides [48]. Notably, the differences in the cytokine levels observed between HER2^- ^and HER2^+ ^cases were independent of the clinical-pathological features showed by the two cohorts of patients (Table 3).

The different immunologic profile of patients with HER2^- ^and HER2^+ ^tumors highlights the importance of considering them as two distinct populations not only with regard to tumor characteristics, but also concerning their immune status. Our analysis, however, may show some limitations mainly due to the quite large number of immunological factors considered. This multiparametric approach has considerably restricted the global case series, thus limiting the possibility to make comparisons of adequate statistical power between subpopulations of HER2^- ^and HER2^+ ^patients. Larger series should be therefore investigated to conclusively rule out the possible influence of distinct clinico-pathological variables on the immunological correlations observed. Moreover, the research of TAA memory T-cell responses was confined at the peripheral immune compartment; an *in situ *comparative survey is needed to confirm our data. An interesting issue that needs further investigation is the assessment of whether the higher percentage of effector memory CD8^+ ^T cells observed in HER2^+ ^patients correlates with the enhanced response against TAA noticed in this population. In perspective, therefore, the characterization of relevant immune parameters may also have a prognostic value, as recently emphasized by the finding that a decreased expression of immune response-associated genes was associated with poor prognosis, particularly in HER2^+ ^cases [49].

Accordingly, the apparently preserved immunological capacity of HER2^+ ^patients may constitute a favorable milieu for several immune system-based therapies. In this respect, a detailed characterization of the immunological profile at diagnosis may be useful to individualize the most promising therapeutic choice or to further contribute in the therapeutic schedule's design. Furthermore, since some chemotherapeutic drugs display beneficial effects on host immune functions [11,50], our results suggest that a careful immune-monitoring of breast cancer patients during NC may be useful to predict the response to therapy and to obtain a better prognostic definition.

## Conclusions

In conclusion, our data indicate that, compared with HER2^- ^cases, patients with HER2-overexpressing, locally advanced breast cancer show a more limited tumor-related immune suppression. This may account for the clinical benefit achieved in this subset of patients with the use of drugs acting through immune-mediated mechanisms. These findings also provide the rationale for further studies aimed at assessing the possible predictive and/or prognostic role of immune markers in locally advanced breast cancer patients undergoing NC.

## Abbreviations

ADCC: antibody-dependent cell cytotoxicity; APCs: antigen presenting cells; CISH: chromogenic *in situ *hybridization; CTLs: cytotoxic T lymphocytes; ELISPOT: enzyme-linked immunosorbent spot; ER: estrogen receptor; FISH: fluorescence *in situ *hybridization; FITC: fluorescein isothiocyanate; HER-2: human epidermal growth factor receptor-2; GM-CSF: granulocyte macrophage colony-stimulating factor; H&E: hematoxylin and eosin; HR: hormone receptor; IFN: interferon; IG: immunoglobulin; IL: interleukin; LAK: lymphokine activated killer; mam-A: mammaglobin-A; muc-1: mucin 1; NC: neoadjuvant chemotherapy; NK: natural killer cell; PBMCs: peripheral blood mononuclear cells; PBS: phosphate-buffered saline; PC: phycoerythrin-cyanine; PE: phycoerythrin; PgR: progesterone receptor; Tregs: regulatory T cells; TAA: tumor-associated antigens; T_CM_: central memory T cells; T_EM_: effector memory T cells; T_Temra_: terminally differentiated T cells; TGF-β1: transforming growth factor-β1; TNF-α: tumor necrosis factor-α; Trag-3: taxol-resistance associated gene 3; ULN: upper limit of normal.

## Competing interests

The authors declare that they have no competing interests.

## Authors' contributions

EM conceived the study, performed most of immunological experiments and drafted the manuscript. DM participated in the design of the study, performed ELISPOT experiments and contributed to draft the manuscript. ET, GM, SS, and DL collected and analyzed clinical data. EC and KM performed part of the experiments. RT carried out all statistical evaluations. TP and AC performed histopathological diagnosis and immunohistochemistry, and performed data analysis. AV reviewed and approved the final manuscript. DC conceived the study and reviewed the manuscript. RD conceived and designed the study, performed data analysis and interpretation and reviewed the manuscript. All authors read and approved the final version of the manuscript for publication.
